# Characterizing relationships of DTI, fMRI, and motor recovery in stroke rehabilitation utilizing brain-computer interface technology

**DOI:** 10.3389/fneng.2014.00031

**Published:** 2014-07-29

**Authors:** Jie Song, Brittany M. Young, Zack Nigogosyan, Leo M. Walton, Veena A. Nair, Scott W. Grogan, Mitchell E. Tyler, Dorothy Farrar-Edwards, Kristin E. Caldera, Justin A. Sattin, Justin C. Williams, Vivek Prabhakaran

**Affiliations:** ^1^Department of Radiology, University of Wisconsin - MadisonMadison, WI, USA; ^2^Department of Biomedical Engineering, University of Wisconsin - MadisonMadison, WI, USA; ^3^Medical Scientist Training Program, University of Wisconsin School of Medicine and Public Health - MadisonMadison, WI, USA; ^4^Neuroscience Training Program, University of Wisconsin School of Medicine and Public Health - MadisonMadison, WI, USA; ^5^Departments of Orthopedics and Rehabilitation, University of Wisconsin - MadisonMadison, WI, USA; ^6^Department of Kinesiology, University of Wisconsin - MadisonMadison, WI, USA; ^7^Department of Medicine, University of Wisconsin - MadisonMadison, WI, USA; ^8^Department of Neurology, University of Wisconsin - MadisonMadison, WI, USA; ^9^Department of Neurosurgery, University of Wisconsin - MadisonMadison, WI, USA; ^10^Department of Psychiatry, University of Wisconsin - MadisonMadison, WI, USA; ^11^Department of Psychology, University of Wisconsin - MadisonMadison, WI, USA

**Keywords:** DTI, FA, fMRI, motor recovery, stroke rehabilitation, BCI

## Abstract

The relationship of the structural integrity of white matter tracts and cortical activity to motor functional outcomes in stroke patients is of particular interest in understanding mechanisms of brain structural and functional changes while recovering from stroke. This study aims to probe these underlying mechanisms using diffusion tensor imaging (DTI) and fMRI measures. We examined the structural integrity of the posterior limb of the internal capsule (PLIC) using DTI and corticomotor activity using motor-task fMRI in stroke patients who completed up to 15 sessions of rehabilitation therapy using Brain-Computer Interface (BCI) technology. We hypothesized that (1) the structural integrity of PLIC and corticomotor activity are affected by stroke; (2) changes in structural integrity and corticomotor activity following BCI intervention are related to motor recovery; (3) there is a potential relationship between structural integrity and corticomotor activity. We found that (1) the ipsilesional PLIC showed significantly decreased fractional anisotropy (FA) values when compared to the contralesional PLIC; (2) lower ipsilesional PLIC-FA values were significantly associated with worse motor outcomes (i.e., ipsilesional PLIC-FA and motor outcomes were positively correlated.); (3) lower ipsilesional PLIC-FA values were significantly associated with greater ipsilesional corticomotor activity during impaired-finger-tapping-task fMRI (i.e., ipsilesional PLIC-FA and ipsilesional corticomotor activity were negatively correlated), with an overall bilateral pattern of corticomotor activity observed; and (4) baseline FA values predicted motor recovery assessed after BCI intervention. These findings suggest that (1) greater vs. lesser microstructural integrity of the ipsilesional PLIC may contribute toward better vs. poor motor recovery respectively in the stroke-affected limb and demand lesser vs. greater cortical activity respectively from the ipsilesional motor cortex; and that (2) PLIC-FA is a promising biomarker in tracking and predicting motor functional recovery in stroke patients receiving BCI intervention.

## Introduction

Studies have suggested that motor recovery after stroke is related to the structural remodeling of white matter tracts (Liu et al., 2008; Schaechter et al., 2009) and the reorganization of cortical activity (Dijkhuizen et al., 2001; Jaillard et al., 2005; Grefkes et al., 2008) in the ipsilesional and contralesional hemispheres. Little is known, however, about the relationship between the white matter structural integrity and functional cortical activity of the sensorimotor region and how these two factors interact with motor recovery in stroke patients. Therefore, a multimodal assessment of structure-function relationships may provide insights for examining factors influencing stroke recovery. Noninvasive brain imaging methods have been widely applied for understanding brain recovery following stroke. Diffusion tensor imaging (DTI) is one of these imaging methods, which allows for quantitative evaluations of the structural integrity of white matter tracts after a stroke (Werring et al., 2000; Stinear et al., 2007; Yu et al., 2009). DTI-derived measures have been shown as potential biomarkers used for tracking motor impairment (Schaechter et al., 2009; Lindenberg et al., 2010; Sterr et al., 2010; Yeo et al., 2010; Borich et al., 2012; Chen and Schlaug, 2013) and motor recovery (Liang et al., 2009) after stroke. DTI has also been investigated for its prognostic potential, with DTI measures assessed during the acute and sub-acute stages of stroke shown to predict motor impairments observed 1–7 months later (Cho et al., 2007; Koyama et al., 2012, 2013a,b; Groisser et al., 2014). A recent study shows evidence that DTI measures may be used as potentially predictive of individual recovery in stroke patients receiving newer neurorehabilitative therapies, such as transcranial direct current stimulation (Lindenberg et al., 2012). Besides DTI, fMRI is another non-invasive neuroimaging technique that has been used to gain better understanding of the processes of brain functional reorganization accompanying motor recovery after stroke (Calautti and Baron, 2003; Riecker et al., 2010; Garrison et al., 2013; Havsteen et al., 2013; Favre et al., 2014; Zhang et al., 2014). Measures derived from fMRI have also been shown as potential biomarkers to track recovery, with correlations between functional changes and fMRI measures demonstrated with treatments such as Brain-Computer Interface (BCI) therapy (Mukaino et al., 2014), constrain-induced movement therapy (Murayama et al., 2011; Kononen et al., 2012) and motor imagery therapy (Sun et al., 2013).

Fewer studies have taken a multimodal approach to characterize brain recovery after stroke by combining information from both DTI and fMRI measures, with a few attempts existing only as case studies (Jang et al., 2005; Caria et al., 2011). One recent study found that DTI-derived measures correlated more strongly with clinical outcomes than measures derived from fMRI (Qiu et al., 2011) and another study reported both DTI and fMRI derived measures correlated with motor outcomes (Chen and Schlaug, 2013). Correlations have also been identified between DTI and fMRI measures, with greater damage to white matter tracts showing an association with increased bilateral recruitment of motor areas and poorer motor performance in stroke patients (Wang et al., 2012), although there is also evidence that such correlations may be modulated after the functional electrical stimulation (FES) training (Wei et al., 2013).

In this study, one main goal is to investigate the relationship between DTI and fMRI measures and further investigate the relative contribution of each to the tracking and predicting of motor functional recovery in a group of stroke patients with persistent upper extremity impairment receiving BCI therapy. In the majority of stroke patients, the upper extremity is more severely involved than the lower limb, as most strokes occur in the territory of the middle cerebral artery (Shelton and Reding, 2001). Stroke that affects the posterior limb of the internal capsule (PLIC) has been reported to be significantly associated with poor recovery of isolated upper-limb movements (Shelton and Reding, 2001) and overall motor outcomes (Puig et al., 2011). Given the significance of PLIC involved in motor recovery, one specific aim of this study is to evaluate the stroke-induced changes in structural integrity of the PLIC using DTI fractional anisotropy (FA) and to investigate if these changes are related to motor recovery. Corticomotor activation is a fMRI way to examine the “integrity” of corticomotor functions. In this study corticomotor activity is evaluated using motor-task fMRI and quantified by counts of statistically significantly active voxels within the ipsilesional and contralesional motor cortices. Another specific aim of this study is to evaluate the changes in corticomotor activity, and to further examine if these changes are related to motor recovery. Combining both DTI and fMRI analysis, we examine the potential relationship between structural integrity of PLIC and functional integrity of motor cortex, and examine how this relationship interact with motor recovery in patients receiving BCI intervention.

## Materials and methods

### Study design

A permuted-block design accounting for gender, stroke chronicity and severity of motor impairment was used to randomize patients to either a BCI intervention group or a crossover control group. Neuroimaging data and motor outcome assessments were acquired at four time points: before the start of intervention (i.e., pre-intervention), at the midpoint of intervention (i.e., mid-intervention), upon completion of intervention phase (i.e., immediately post-intervention), and 1 month following the last session of BCI intervention (i.e., 1-month-post-intervention). Patients in the BCI intervention group began to receive BCI intervention soon after recruitment. Patients in the control group first received three additional neuroimaging scans and motor outcome assessments during the control phase in which no BCI intervention was administered. These three additional assessments were acquired at intervals analogous to those administered during the BCI intervention phase. Upon completion the final-control neuroimaging and motor outcome assessment, these patients were crossed over to complete the BCI intervention phase. Table 1 illustrates the time frame of the study design. All current findings are based on neuroimaging and motor outcome measurements acquired from 9 patients *during the BCI intervention phase*.

**Table 1:**
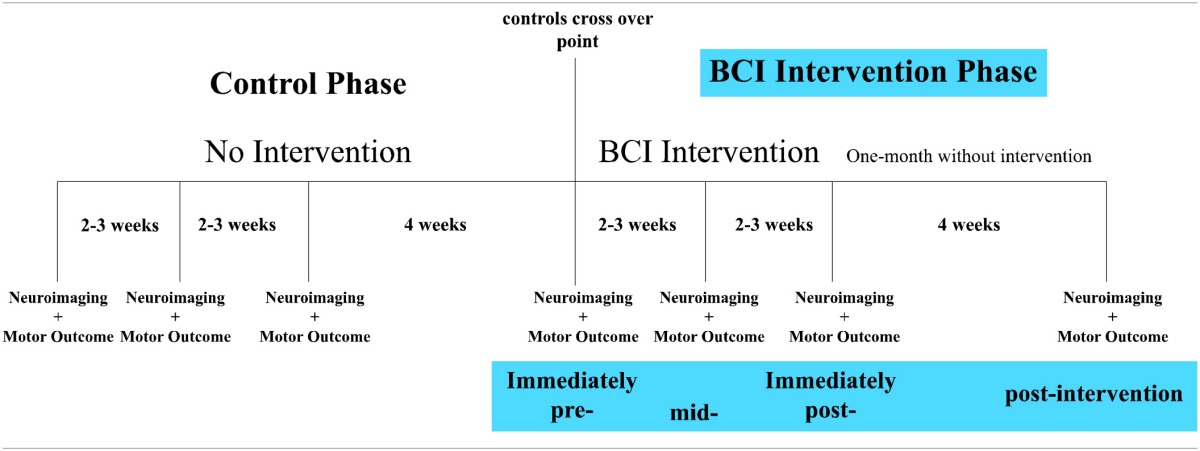
**Study design**.

### Patient characteristics

Sixteen patients with persistent upper extremity motor impairment resulting from first-ever ischemic or hemorrhagic stroke were contacted regarding study participation in an on-going study investigating effects of EEG-BCI driven FES therapy of the impaired hand in stroke patients. This report is based on 9 patients who have completed the study (6 M, mean age of 61.9 years, chronicity of stroke range 2–23 months).

The inclusion criteria were: (1) ages 18 years and above; (2) no known neurologic, psychiatric or developmental disability; (3) persistent upper-extremity motor impairment resulting from ischemic or hemorrhagic stroke. The exclusion criteria were: (1) contraindications for MRI; (2) allergy to electrode gel, surgical tape and metals that would be used in BCI intervention; (3) under treatment for infectious disease or having apparent oral lesions or inflammation. This study was approved by the University of Wisconsin-Madison's Health Sciences Institutional Review Board. All patients provided written informed consent. Patient profiles are shown in Table 2.

**Table 2:** **Patient profiles (age, gender, time since stroke, baseline NIHSS, baseline NIHSS-motor arm and stroke location)**.

**Subject ID**	**Age**	**Gender**	**Months since stroke**	**Baseline NIHSS**	**NIHSS-motor arm**	**Stroke location**
CI001	52	M	15	8	4	Left MCA
CI002	62	F	16	8	4	Left precentral gyrus
CI003	68	M	3	0	0	Left frontal lobe
CI004	66	M	23	6	1	Left MCA
CI005	73	F	2	0	0	Left MCA
CT001	75	F	23	7	3	Right putamen
CT002	55	M	17	0	0	Left basal ganglia
CT003	49	M	6	3	1	Right pons
CT004	57	M	13	2	1	Left MCA
**Mean ± SD**	**61.89 ± 9.25**	**3F/6M**	**13.11 ± 7.90**	**3.78 ± 3.49**	**1.56 ± 1.67**	**3 sub-cortical**

*MCA, Middle Cerebral Artery*.

### BCI intervention procedures

All patients were administered up to 15 two-hour sessions of interventional BCI therapy (13.11 ± 2.20 sessions). Figure S1 illustrates a conceptual schematic of the system. A detailed description of the procedures followed during each session is provided in the Supplementary Material. These sessions took place over a period of up to 6 weeks with two to three intervention sessions per week.

### Motor functional outcome measures

All patients were assessed for clinical stroke severity in addition to neurologic examination at four time points throughout the intervention (Table 1). The neurologic deficit was evaluated on the basis of the severity of motor paresis using the National Institute of Health Stroke Scale (NIHSS) (Brott et al., 1989). All patients' motor function of the impaired arm was assessed using a neuropsychological battery which included objective measures such as the Action Research Arm Test (ARAT) (Carroll, 1965; Lang et al., 2006) and subjective measures such as the Stroke Impact Scale-Hand function domain (Duncan et al., 1999). The ARAT is a standardized measure of upper-limb functioning assessing grip, grasp, pinch and gross motor performance. Total ARAT scores ranged from 0 to 57. The Stroke Impact Scale (SIS) hand function subscale (SIS-Hand) was used to assess self-reported satisfaction with hand use and to evaluate the relationship between SIS-Hand scores and neuroimaging measures. Raw scores of SIS-Hand assessment were transformed using the following algorithm (Sullivan, 1995): Transformed scale = 100 × [(actual raw score − lowest possible raw score)/possible raw score]. The transformed scale of SIS-Hand score ranged from 0 to 100. All clinical assessments of the stroke-affected limb for each patient at each time point are shown in Table S3.

### Neuroimaging data

Neuroimaging data acquisition and processing are described in detail in the Supplementary Material. FA values were computed for the ipsilesional and contralesional PLIC. In addition, asymmetry indices between the ipsilesional and contralesional PLIC-FA (aFA) were calculated as aFA = (FA_contra_ − FA_ipsi_)/(FA_contra_ + FA_ipsi_) (Stinear et al., 2007; Schaechter et al., 2009; Lindenberg et al., 2010). This yields a value of aFA ranging from −1.0 to +1.0 with positive values indicating reduced FA in the ipsilesional PLIC, a value of 0 indicating symmetrical FA measurements from the two hemispheric PLIC, and a negative value indicating reduced FA in the contralesional PLIC.

All patients performed a block-design sequential finger tapping task during fMRI scans that consisted of alternating 20-s blocks of tapping vs. rest. Patients were cued to rest or to tap the fingers of one hand sequentially on a button box, using either visual or tactile (for visually impaired patients) cues. All patients underwent two fMRI scans using this paradigm –once when tapping with the impaired hand (passive tapping if unable to generate sufficient tapping independently) and again when tapping with the unimpaired hand. Patients were instructed to hold their heads still throughout the scans, and sufficient padding was provided to discourage head movement.

In the passive motor tasks, patients were assisted by the investigator in finger movements (flexion-extension) to complete the finger tapping tasks according to the experimental paradigm design.

### Motor-task generated active voxels

A previously published mask of the cortical components of the motor network was used to identify statistically significantly active voxels in the motor cortex during finger tapping. This mask consisted of the cortical components of a previously identified motor network derived from an independent component analysis (ICA) of whole-brain resting-state fMRI (rs-fMRI) scans (Shirer et al., 2012). These independent components were visually selected based on previous reports and then thresholded independently and arbitrarily to generate distinct moderately sized ROIs in the cortex and subcortical gray matter (voxels = 25) (Shirer et al., 2012). It is worth noting that although rs-fMRI investigates synchronous activations between brain regions occurring in the absence of a task or stimulus, these synchronous activations have been observed in somatosensory, visual, attention and other higher-order brain areas and have shown close correspondence between the independent analyses of resting and activation brain dynamics (Biswal et al., 1995; Smith et al., 2009). In our study, only the cortical components from the motor network for each hemisphere were used for evaluation of corticomotor activity. Once fMRI data was processed (see Supplementary Material), this motor cortical mask was resampled into subject space and then applied to the functional data to identify those statistically significantly active voxels within the motor cortex using a threshold of *t* = 4 (*p* < 0.0001).

### Statistical analysis

Considering the relatively small sample size (*n* = 9), we used non-parametric statistical tests for the analyses. The Wilcoxon signed-rank test was used to compare ipsilesional and contralesional PLIC FA values and to compare corticomotor fMRI activity between the two motor cortices. The Spearman rank correlation test was used for analyses of neuroimaging and motor outcome measurements. To take advantage of a longitudinal, repeated-measurement design of this study, we used generalized estimating equations (GEE) for regression analyses. GEE analyses use the generalized linear model to estimate more efficient and unbiased regression parameters relative to ordinary least squares regression (Ballinger, 2004). Most importantly, the GEE analyses take into account the dependency of repeated measurements from the same patient in the regression analysis. In addition, ANOVAs were used to examine how factors of time (pre-, mid-, immediately post-, and 1-month-post), PLIC (contralesional vs. ipsilesional side) and interaction between time and PLIC affecting DTI and fMRI measures. All statistical analyses were performed using RStudio (version 0.97.318). A *p* value less than or equal to 0.05 was considered statistically significant.

## Results

### Patient characteristics and clinical measures

Patient characteristics are summarized in Table 2. Average age was 61.89 years (*SD* = 9.25 years); average time from stroke onset was 13.11 months (*SD* = 7.90 months). There were no significant differences in terms of left or right hemisphere stroke (*p* = 0.14), cortical or non-cortical stroke (*p* = 0.36), or gender (*p* = 0.36). Standard clinical MRI was used to assess damage to PLIC by the neuroradiologist Dr. Prabhakaran. Six of the nine patients showed damage to PLIC due to stroke. Patient CT004 with a left middle cerebral artery (MCA) territory infarct showed minimal damage to PLIC. Patients CI003 with a small left frontal lobe infarct and CT003 with a right pontine infarct did not show damage to PLIC.

Clinical motor outcome measures are summarized in Table S3. The ARAT scores varied from zero, indicating no ability to perform, to a maximum of 57, indicating unimpaired performance. The SIS measure of hand function varied widely, with a value of zero indicating a patient reporting no ability to use the impaired hand, and higher positive values indicating decreasing levels of difficulty using the impaired hand in daily activities such as carrying heavy objects, turning a doorknob, opening a can or jar, tying a shoe lace and picking up a dime.

### Relationship of PLIC-DTI measures and motor outcomes

**Figure 1:**
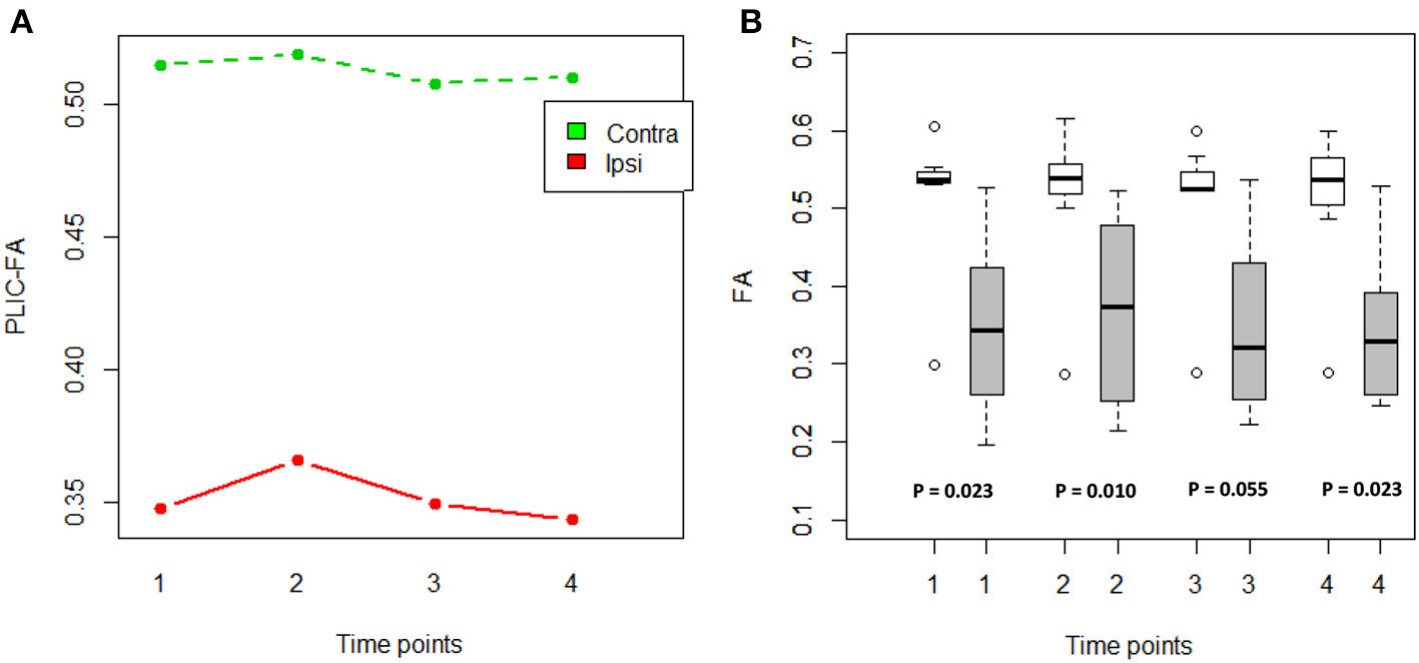
**FA values compared between the ipsilesional and contralesional sides of the PLIC. (A)** PLIC-FA changes across time compared between two hemispheres using ANOVA tests. Contra, contralesional PLIC; Ipsi, ipsilesional PLIC. **(B)** Boxplots showed significantly lower FA in ipsilesional PLIC when compared to contralesional PLIC (Time of pre-, mid-, post-, and 1-month-post intervention are indicated as time-point 1, 2, 3, and 4, respectively). White boxes represent the contralesional side and gray boxes represent the ipsilesional side of PLIC. (Wilcoxon signed-rank tests; all *p*-values are listed).

An ANOVA was computed to examine the main effects of time and PLIC (contralesional vs. ipsilesional side) as well as time × PLIC interaction (Figure 1A). Only PLIC factor was significant (*p* = 5.56e-07), and this was further validated with a Wilcoxon signed-rank test. Ipsilesional PLIC FA values were significantly lower when compared to the contralesional side (Wilcoxon signed-rank test: *p* < 0.05) except at time point 3 (immediately post-intervention) with a *p*-value equal to 0.055 trending toward significance (Figure 1B).

**Figure 2:**
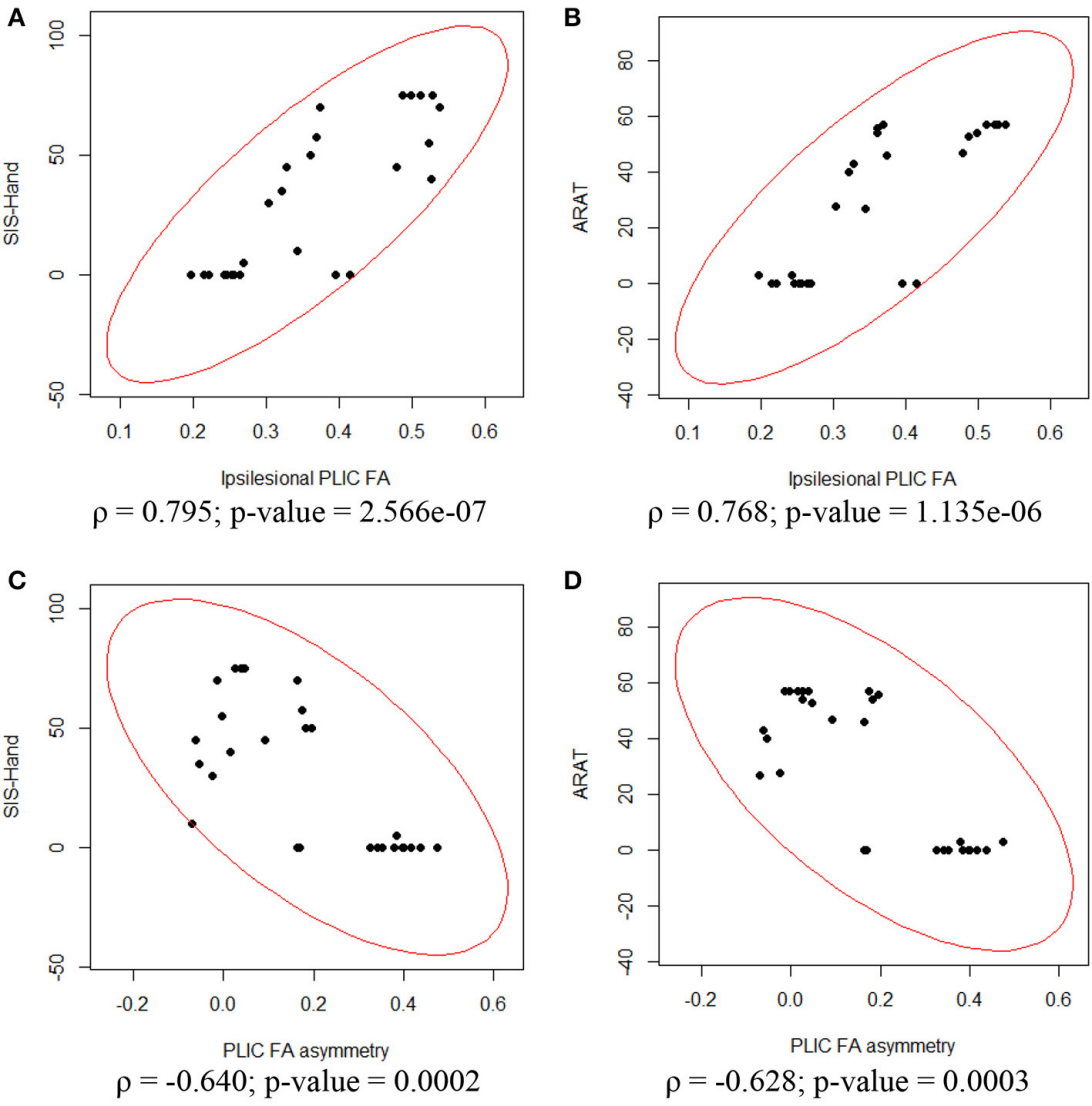
**Correlation analyses between ipsilesional FA, aFA and motor outcomes assessed in the impaired hand**. Spearman rank correlation tests showed significant relationships between DTI and motor outcome measurements. **(A)** Ipsilesional PLIC-FA was positively correlated with SIS-hand function. **(B)** Ipsilesional PLIC-FA was positively correlated with ARAT. **(C)** PLIC-aFA was negatively correlated with SIS-hand function. **(D)** PLIC-aFA was negatively correlated with ARAT.

**Figure 3:**
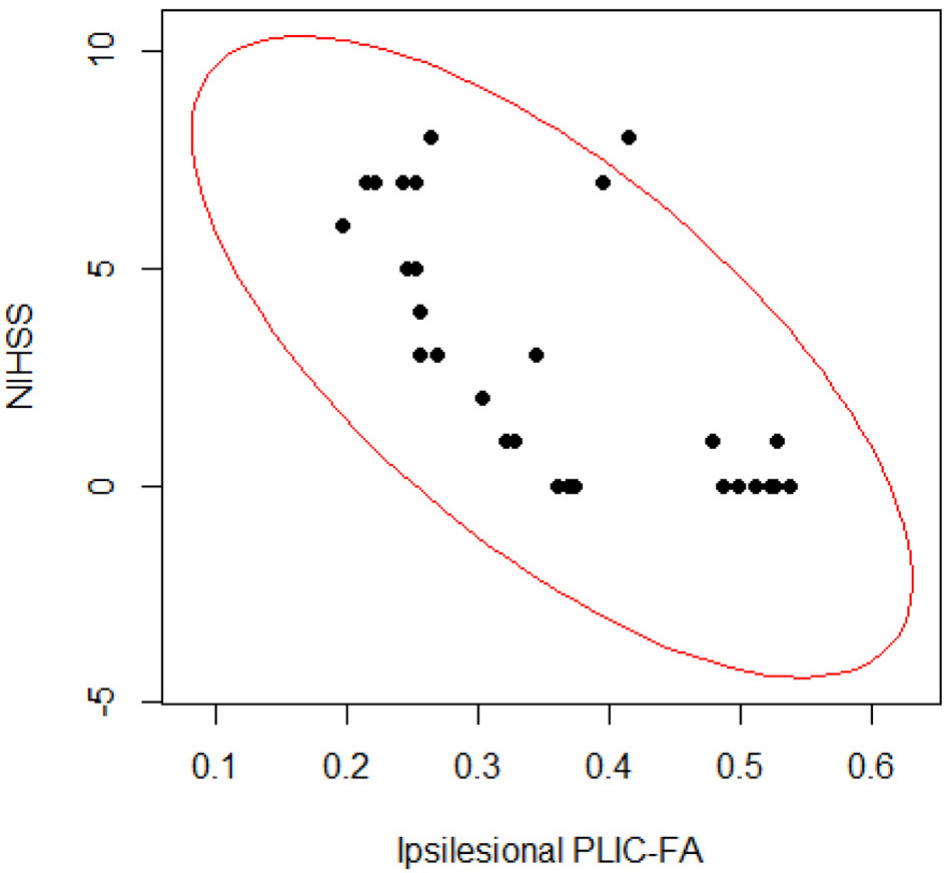
**Correlation analyses on stroke severity (NIHSS scores) and structural integrity of PLIC (FA values)**. The Spearman rank correlation test (ρ = −0.695; *p*-value = 2.892e-05) and GEE regression analysis (GEE *p*-value = 3.691e-04) showed significant relationships between PLIC-FA values and patients' clinical severity assessments (NIHSS scores).

To assess the relationship between PLIC-FA values and motor outcome measures, a Spearman rank correlation test was first performed on the longitudinal data acquired from all patients and from all time points. The results suggested that higher ARAT scores and higher SIS-Hand scores were significantly correlated with higher FA values in ipsilesional PLIC (Figure 2). PLIC FA asymmetry (aFA) was negatively correlated with ARAT and SIS-Hand scores (Figure 2). A secondary statistical analysis, the GEE analysis, was further computed to control for the dependence of repeated measurements from each patient across time (Table 3). This analysis confirmed that the relationship observed between PLIC-FA and motor outcomes remained statistically significant. In addition, stroke severity as assessed by the NIHSS was significantly and negatively correlated with ipsilesional PLIC-FA values (Figure 3; GEE regression coefficient = −33.57, *p*-value = 3.69e-04).

**Table 3:** **Correlation analyses of DTI, fMRI and motor recovery measures**.

**(A) SPEARMAN RANK CORRELATION TESTS ON NEUROIMAGING AND MOTOR OUTCOME MEASURES**				
**Motor outcomes**	**Spearman rank correlation test**	**FA**	**aFA**	**Voxel counts**
SIS-Hand	correlation coefficient	0.795	−0.640	−0.463
	*p*-value	2.566e-07	0.0002	0.012
ARAT	correlation coefficient	0.768	−0.628	−0.414
	*p*-value	1.135e-06	0.0003	0.026
**(B) GEE REGRESSION ANALYSES BETWEEN NEUROIMAGING AND MOTOR OUTCOME MEASURES**				
**Motor outcomes**	**GEE**	**FA**	**aFA**	**Voxel counts**
SIS-Hand	Regression coefficient	229.89	−138.47	−0.01
	*p*-value	**1e-06**	**6.86e-10**	0.55
ARAT	Regression coefficient	127.93	−116.50	−0.03
	*p*-value	**0.001**	**6.68e-09**	0.27

### Prediction of motor function recovery with baseline neuroimaging measures

**Figure 4:**
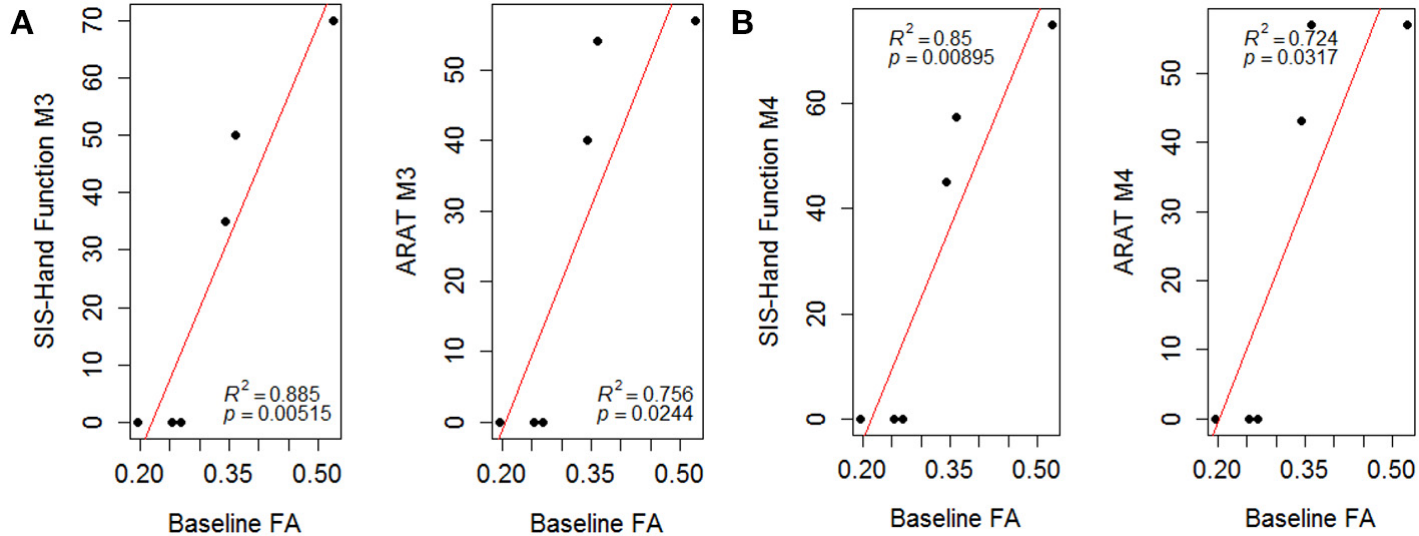
**Scatter plots of PLIC-FA predicting motor outcomes evaluated (A) immediately post-intervention (M3) and (B) 1-month-post intervention (M4)**. Linear regression analyses demonstrated that baseline PLIC-FA can predict motor outcomes. Pearson's correlation coefficients (ρ) and r-squared (*R*^2^) values are shown on the figures.

The linear regression analyses using a least-square fitting method revealed that baseline ipsilesional PLIC-FA correlated with the post-intervention ARAT and SIS-Hand scores (Figure 4; r-squared values > 0.7). Ipsilesional PLIC FA values measured at pre-intervention (baseline) were significantly and positively correlated with motor outcome scores measured immediately post- and 1-month-post intervention (*p*-value = 0.05). The same approach was applied to fMRI voxel counts, which did not reveal a predictive relationship with this fMRI measure on motor recovery.

### Relationship of corticomotor activity and motor outcomes

**Figure 5:**
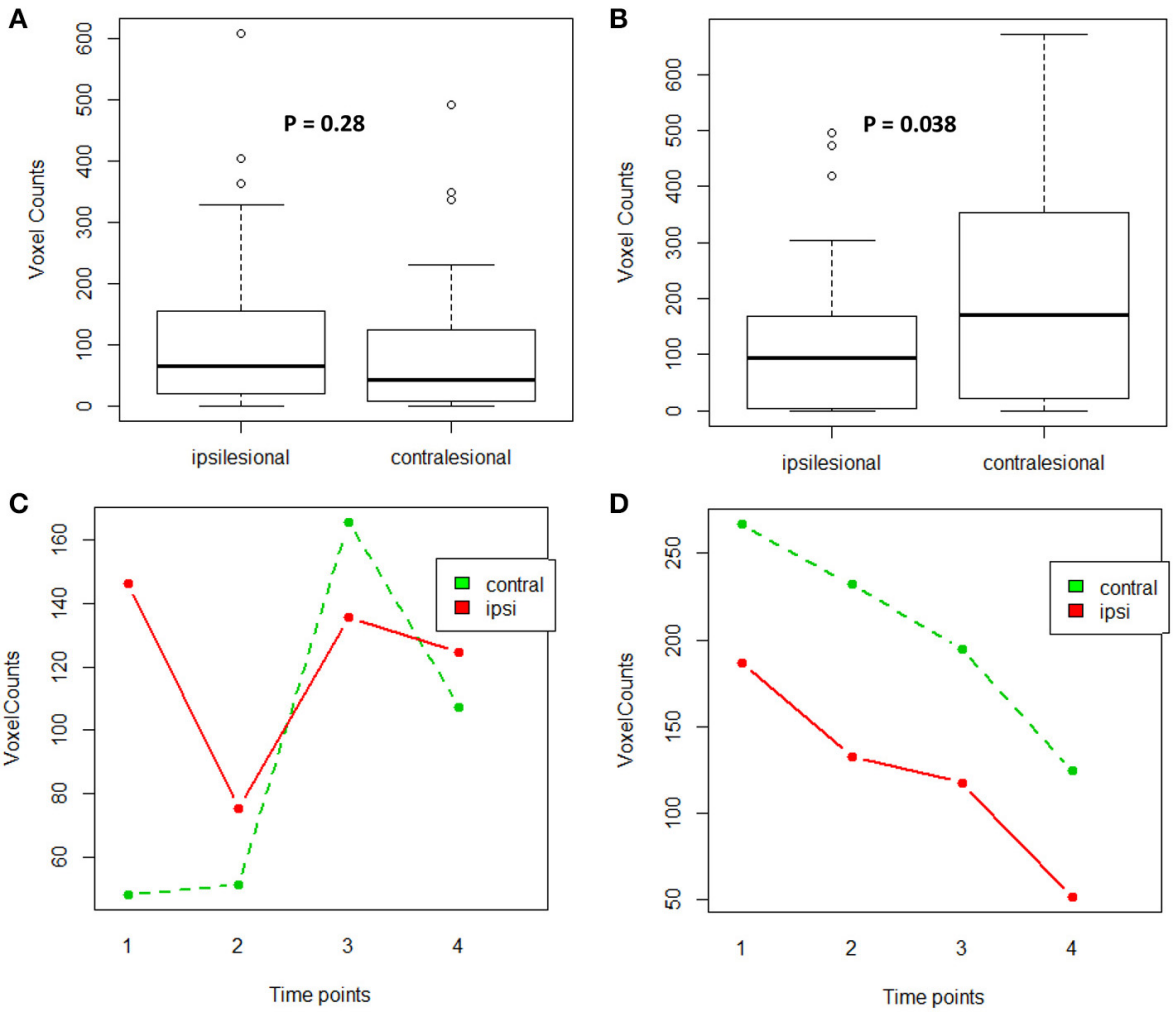
**Comparison of active voxel counts during impaired and unimpaired finger tapping during fMRI scans**. **(A)** Impaired finger tapping condition examining lesion factor, **(B)** Unimpaired finger tapping condition examining lesion factor, **(C)** Impaired finger tapping condition examining both lesion and time factors, **(D)** Unimpaired finger tapping condition examining both lesion and time factors.

For the impaired finger tapping task, counts of active voxels were not significantly different between the two motor cortices (Wilcoxon signed-rank test: *p* = 0.28) (Figure 5A). Note, passive motor-task fMRI data was collected for patients CI001, CI002, CI004, CT001, and CT003 who were unable to perform motor tasks during fMRI scans. For the unimpaired finger tapping task, active voxel counts were significantly greater within contralesional motor cortex compared with the ipsilesional side (Wilcoxon signed-rank test: *p* = 0.038) (Figure 5B). An ANOVA was computed to examine the effects of time and PLIC (contralesional vs ipsilesional side) and the time × lesion interaction for both fMRI measures from impaired and unimpaired finger tapping. The results revealed no significant changes in corticomotor activity due to any of these factors (Figures 5C,D). However, the influence of PLIC trended toward significance (*p* = 0.064) for the unimpaired finger tapping task.

Given the changes observed in corticomotor activity across time for impaired finger tapping compared to unimpaired finger tapping, another ANOVA was computed to test the effects of time (pre-, mid-, immediately post- and 1-month-post), PLIC (contralesional vs. ipsilesional), hand-impairment (impaired vs. unimpaired), and interaction between time and hand-impairment. We found that both the effect of hand-impairment and interaction between time and hand-impairment were significant (*p*-value = 0.005 and 0.015 respectively).

The Spearman rank correlation tests also demonstrated that fMRI measures (i.e., active voxel counts in ipsilesional motor cortex generated from impaired finger tapping task) were associated with motor outcomes. This negative association between motor outcomes and fMRI measurements may suggest that better motor outcomes after BCI-intervention are associated with a smaller number of active voxels within the ipsilesional motor cortex (Figure S2). However, this relationship was no longer significant after GEE regression analyses accounted for the dependence of repeated measurements from each patient (Table 3).

### Relationship between DTI and fMRI measurements

**Figure 6:**
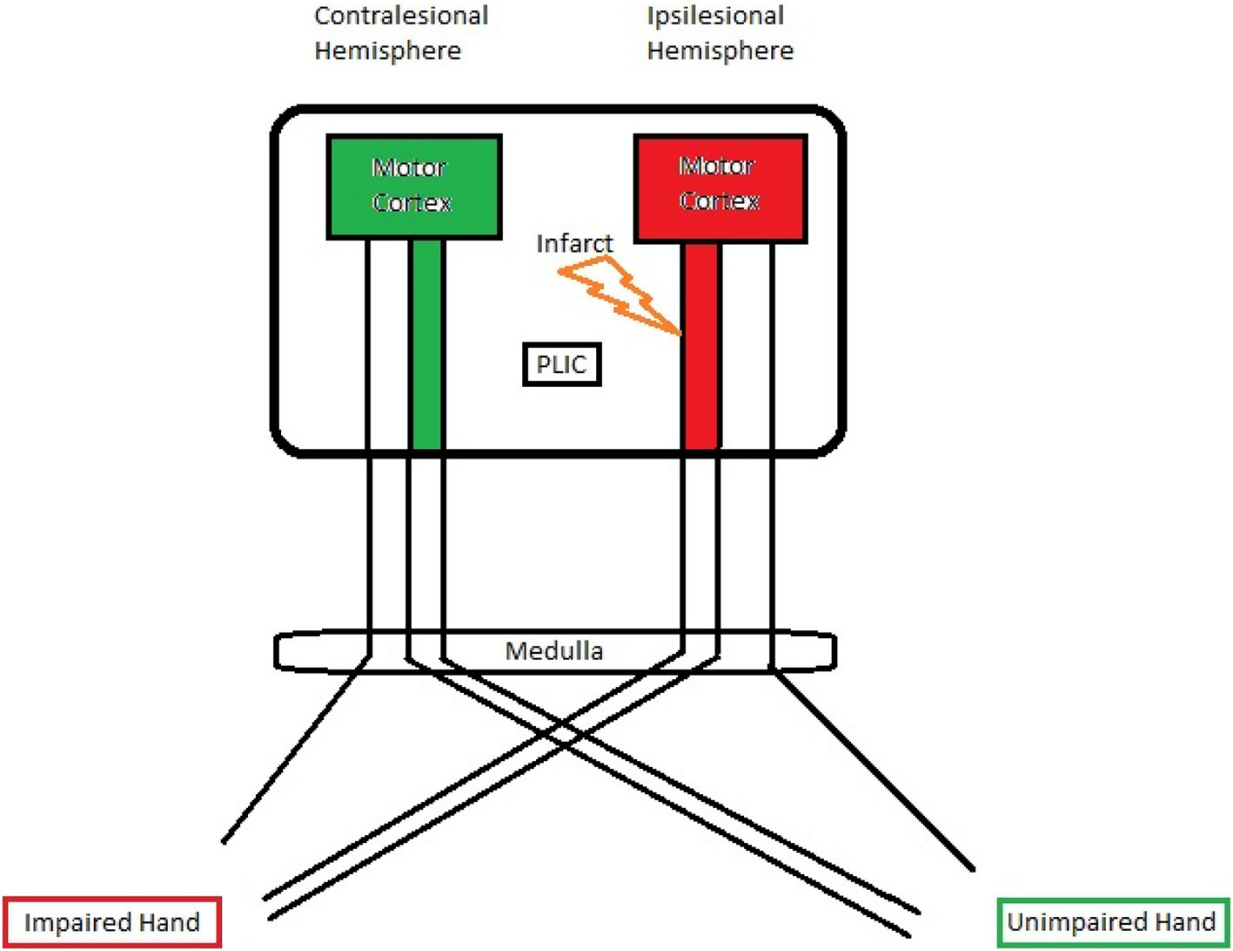
**A schematic illustration of the PLIC and motor cortex**. A significant negative correlation was observed between ipsilesional PLIC-FA and ipsilesional motor activity.

The corticospinal pathway from the primary motor cortex through the PLIC approaches the midline of lower medulla oblongata and crosses to the contralateral side at the pyramidal decussation (Vulliemoz et al., 2005). A small percentage of fibers (10–25%), however, remain ipsilateral (Davidoff, 1990). A schematic illustration of the PLIC and motor cortex is shown in Figure 6. Taking this fact into account, we examined the DTI-fMRI relationship within each hemisphere for both impaired and unimpaired finger tapping tasks. The Spearman rank correlation test followed by GEE was used for correlation analyses.

**Figure 7:**
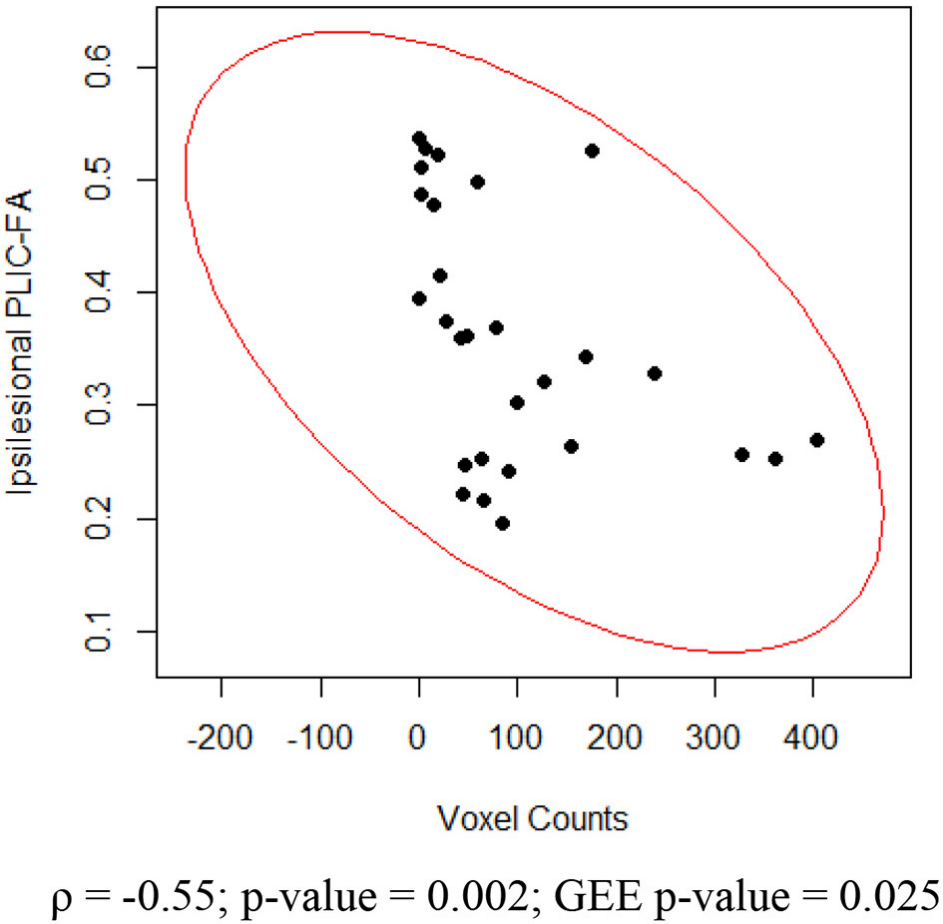
**Relationship between ipsilesional corticomotor activity and ipsilesional PLIC-FA**. Shown here are Spearman rank correlation coefficient (ρ) and corresponding *p*-value as well as GEE *p*-value.

For the impaired finger tapping, crossing fibers form the majority of ipsilesional PLIC, and may be affected by the small percentage of PLIC fibers on the contralesional side (Vulliemoz et al., 2005). We found that ipsilesional PLIC-FA negatively correlated with active voxel counts within the ipsilesional motor cortex (Figure 7). We did not, however, observe a significant relationship between contralesional PLIC-FA and voxel counts within the contralesional motor cortex. A similar approach was applied for measurements from unimpaired finger tapping. No significant relationship was found between PLIC-FA and voxel counts within ipsilesional or contralesional hemisphere during unimpaired finger tapping.

## Discussion

Much of the focus of fMRI in stroke studies has been on whether its application provides a better understanding of brain functional reorganization accompanying motor recovery after stroke (Riecker et al., 2010; Garrison et al., 2013; Havsteen et al., 2013; Heiss and Kidwell, 2014). In a recent study, fMRI-derived measures have been correlated with movement recovery achieved with robot-assisted BCI therapy (Varkuti et al., 2013), while another study found that fMRI measures do not contribute significantly to the prediction of motor recovery (Zarahn et al., 2011). Another non-invasive MRI-based technique, DTI, has been widely used to evaluate the integrity of the white matter tracts after stroke. DTI-derived measures have been shown to be potential biomarkers for tracking motor impairment (Lindenberg et al., 2010; Chen and Schlaug, 2013) and motor recovery (Liang et al., 2009) after stroke. This study investigated the relationship between white matter integrity evaluated by DTI FA at the PLIC and corticomotor activity measured by motor-task fMRI, and further examined if these imaging measurements correlate with motor functional recovery in stroke patients receiving a BCI-facilitated intervention. Although our findings are preliminary and based on a moderate-size dataset, we observed consistent and robust results which are discussed here.

### Structural integrity of the PLIC vs. motor recovery

Previous human and animal studies have characterized changes in FA in the corticospinal system due to stroke (Liu et al., 2007; Kusano et al., 2009; Schaechter et al., 2009; Lindenberg et al., 2012). In our study, DTI analyses on 9 stroke patients with varying lesion locations and size of infarct affected the corticospinal system and yielded consistent observations, specifically, decreased FA in the ipsilesional PLIC compared to the contralesional side. This has been suggested as a characteristic of chronic white matter “Wallerian” degeneration (Yu et al., 2009; Lindenberg et al., 2012) and is thought to arise from the loss of tissue structural integrity (Liu et al., 2007).

In the current study, we found that higher ARAT and SIS-Hand scores were significantly correlated with higher FA values measured within ipsilesional PLIC after accounting for repeated measurements (Table 3). To account for potential changes in contralesional PLIC, FA asymmetry between the ipsilesional and contralesional sides were calculated (Stinear et al., 2007; Lindenberg et al., 2010) and then correlated with motor outcome measurements. The relationship between FA asymmetry and motor outcomes was significant even when accounting for repeated measurements. Lower or near zero aFA values indicate better preserved integrity of the ipsilesional PLIC and were correlated with better motor outcomes. Given our observation that the ipsilesional PLIC had decreased FA compared with the contralesional side, these findings suggest that the more the ipsilesional PLIC-FA resembled the contralesional PLIC, the greater the potential for functional recovery in stroke-affected limb.

### Predictive value of the PLIC-FA in motor recovery

One novel finding of this study is the predictive value of PLIC-FA in predicting recovery of motor function. Baseline FA values were significantly correlated with motor outcomes measured after intervention both immediately post-intervention and 1-month-post intervention (Figure 4). This suggests PLIC-FA may be useful as a novel biomarker to predict upper-limb motor functional recovery of stroke patients receiving BCI interventions.

### Corticomotor activity

For impaired finger tapping, we observed a bilateral pattern of corticomotor activity (Figure 5A), while unimpaired finger tapping produced significantly more lateralized activity within the contralesional motor cortex in comparison to ipsilesional motor cortex (Figure 5B). When comparing corticomotor activity for impaired and unimpaired finger tapping, we observed a significant difference due to the factor of hand-impairment (ANOVA test, *p*-value = 0.005). Furthermore, the pattern of changes in voxel counts (i.e., interaction between factors of time and hand-impairment) was also significant between the two tapping conditions (ANOVA test, *p*-value = 0.015). These findings may suggest that a potential bilateral pattern of corticomotor activity evolved during motor recovery in these stroke patients receiving BCI intervention.

### Corticomotor activity vs. structural integrity of the PLIC

Our results are that greater stroke severity is significantly correlated with compromised PLIC in the ipsilesional hemisphere (Figure 3). Furthermore, the ipsilesional PLIC-FA values are negatively correlated with ipsilesional corticomotor activity during impaired finger tapping (Figure 7). Thus, a greater burden of cortical activation is placed on the ipsilesional motor cortex for impaired finger tapping in patients with greater stroke severity with an overall bilateral pattern of motor cortical involvement. The BCI intervention may contribute to the increased utilization of ipsilesional and contralesional motor cortex with more bilateral activity seen during impaired finger tapping, rather than the lateralized activity seen normally during unimpaired finger tapping (Figures 2A,B). These factors may ultimately place demands on both ipsilesional and contralesional motor cortices during stroke recovery. This is novel in comparison to previous studies that suggest successful therapeutic intervention produces restoration of motor function mediated by re-lateralization of motor cortical activation (Ward et al., 2003a,b; Saur et al., 2006; Schlaug et al., 2008).

### Limitations

The small sample size (*n* = 9) and the heterogeneity of stroke patients (Table 2) were the primary limitations of this study. Four of the 9 patients exhibited no or little improvement in functional recovery as assessed by clinical behavioral performance. Those patients were severely impaired and minimally able to perform the designed intervention tasks, resulting in a floor effect in some outcome measurements. While changes in fMRI and DTI measurements were observed across time, an ANOVA did not show these changes to be significant, which may also be due to small sample size and high between-patient variance. It is also worth noting that our current findings are preliminary and are based on a moderate-size dataset.

Another limitation of our study is the combined analysis of passive and active finger tapping tasks performed by these patients. Passive vs. active tasks may have different effects on corticomotor activity which was further described and discussed in Supplementary Material.

Current DTI techniques remain limited in their ability to untangle the mix of PLIC fibers from the ipsilateral and contralateral hemispheres as they descend along the corticospinal pathway. Although we used task fMRI with each hand to investigate the structure-function relationship of the PLIC and the motor cortex, the current study design does not allow us to separate the mixture of white matter tracts from ipsilateral and contralateral corticospinal pathways within the PLIC which constrains our current findings. Future studies may therefore need to be done utilizing high resolution DTI and tractography techniques to further investigate the relationship between structural and functional changes in stroke patients.

## Author contributions

Jie Song assisted with subject recruitment, data collection, data analysis, and writing. Brittany M. Young assisted with subject recruitment, data collection, data analysis, and writing. Zack Nigogosyan assisted in data collection and data analysis. Leo M. Walton assisted with data collection. Veena A. Nair assisted with subject recruitment, data collection, data analysis, and writing. Scott W. Grogan assisted with data collection and data analysis. Mitchell E. Tyler provided TDU hardware and expertise. Dorothy Farrar-Edwards assisted with study design and outcome measure selection and interpretation. Kristin E. Caldera assisted with subject recruitment. Justin A. Sattin assisted with study design and subject recruitment. Justin C. Williams is one of two lead PI's on this project and supervised the technical and engineering aspects of the work. Vivek Prabhakaran is one of two lead PI's on this project and supervised the neuroimaging aspects of this work.

### Conflict of interest statement

There is one patent pending on the closed-loop neurofeedback device used for the BCI-facilitated intervention administered in this study (Pending U.S. Patent Application No. 12/715,090). This patent was filed jointly by the two lead investigators Justin C. Williams and Vivek Prabhakaran. The authors declare that the research was conducted in the absence of any commercial or financial relationships that could be construed as a potential conflict of interest.
