# Supplementary material for: Characterizing relationships of DTI, fMRI, and motor recovery in stroke rehabilitation utilizing brain-computer interface technology
Source: Front Neuroeng. 2014 Jul 29;7:31. doi: 10.3389/fneng.2014.00031 (PMC4114288; doi:10.3389/fneng.2014.00031)
Supplement: Supplementary file 1 [file DataSheet1.DOCX]

**Supplementary Material:**

Characterizing Relationships of DTI, fMRI, and Motor Recovery in Stroke Rehabilitation Utilizing Brain-Computer Interface Technology

**Authors:**

J. Song^1, 2^, B.M. Young^1, 3, 4^, Z. Nigogosyan^1^, L.M. Walton^2, 4^, V.A. Nair^1^, S.W. Grogan^1^, M.E. Tyler^2,8^, D.F. Edwards^5-7^, K.E. Caldera^8^, J.A. Sattin^7^, J.C. Williams^2,4,9^, V. Prabhakaran^1, 3, 4, 7,10,11^

**Affiliations:**

^1^ Department of Radiology, University of Wisconsin - Madison, Madison, WI. USA.

^2^ Department of Biomedical Engineering, University of Wisconsin - Madison, Madison, WI. USA.

^3^ Medical Scientist Training Program, University of Wisconsin - Madison, Madison, WI. USA.

^4^ Neuroscience Training Program, University of Wisconsin - Madison, Madison, WI. USA.

^5^ Departments of Kinesiology, University of Wisconsin-Madison, Madison, WI, USA.

^6^ Departments of Medicine, University of Wisconsin-Madison, Madison, WI, USA.

^7^ Department of Neurology, University of Wisconsin – Madison, Madison, WI, USA.

^8^ Department of Orthopedics and Rehabilitation, University of Wisconsin – Madison, Madison, WI, USA.

^9^ Department of Neurosurgery, University of Wisconsin – Madison, Madison, WI, USA.

^10^ Department of Psychiatry, University of Wisconsin – Madison, Madison, WI, USA.

^11^ Department of Psychology, University of Wisconsin – Madison, Madison, WI, USA.

**BCI Intervention Procedures**

Subjects participating in the study were administered up to 15 sessions of BCI intervention, each session lasting for approximately 2 hours with a few minutes of break if desired. Figure S1 illustrates a conceptual schematic of the training system.

In the beginning of intervention, subjects underwent screening and were asked to perform attempted and imagined hand movement of each side alternating with periods of rest. Cues for movement and rest were presented as words (‘right’, ‘left’ and ‘rest’) on a computer screen that was placed at a comfortable visual distance in front of the subject. Each cue was presented for a period of 4 seconds. While subjects undergoing screening, EEG was recorded and analyzed off-line to identify features (i.e., μ (8–12 Hz) and β (18–26 Hz) rhythms) of cortical activity associated with attempted and imagined hand movement.

Following screening, subjects were instructed to perform BCI control tasks. In BCI control tasks, subjects were asked to perform intended hand movement in order to hit a target rectangle with a cursor ball. The target was presented on the right or left side of the computer screen and the cursor ball moved horizontally. Subjects with residual hand function in affected hand would perform actual movement. Subjects with less function in their affected hand would perform intended or imagined movement. Cortical activity related to subject’s hand movement as detected by EEG was translated into one-dimensional movement of the cursor ball, which was presented on the computer screen as real-time feedback provided by the BCI system, allowing subjects to learn how to modulate their cortical activity accordingly. A trial was considered as a success only if the subject was able to maneuver the cursor ball to hit the target within 5 seconds. A typical 2-hour session of BCI intervention consisted 20 to 40 runs, with 10 to 12 trails in each run.

Once subjects achieved consistent accuracy (i.e., over 70% successful rate) in doing this task, FES was added to muscles of the affected arm to assist with the actual or intended movement. FES was programed to be triggered only when the subject was cued to perform movement using the affected hand and cortical activity related to affected-hand movement was detected by the system.

Additional tactile feedback provided via a tongue display unit (TDU) [1] was also incorporated into the BCI system. TDU was controlled to provide continuous electro-tactile stimulation of the tongue in parallel with the visual feedback within each trial. Tactile feedback coded the current position of the target and cursor into electrical stimulus which was delivered onto the subject’s tongue by TDU. In this study, subjects CI001, CI002, CI003 and CT004 obtained full sessions of tongue stimulation during the course of intervention.

**Neuroimaging data acquisition**

Both MRI and DTI data were obtained on a 3.0 Tesla whole-body MRI scanner (GE DISCOVERY MR750) with an 8-channel head coil. A T1-weighted high-resolution anatomical scan was obtained for each subject using a BRAVO FSPGR pulse sequence before fMRI and DTI data acquisition. Imaging parameters for the anatomical T1-weighted scan are: TR = 8.16ms, TE = 3.18ms, flip angle = 12º, field of view (FOV) = 256 × 256 mm^2^, matrix size = 256 × 256, and 156 axial slices of 1mm thickness. MRI data were acquired using a T2*-weighted gradient-echo EPI pulse sequence sensitive to BOLD contrast. Imaging parameters are as follows: TR = 2600ms, TE = 22ms, flip angle = 60º, FOV = 224 × 224 mm^2^, matrix size = 64 × 64, and 40 axial slices of 3.5mm thickness. Diffusion imaging parameters are: single shot EPI sequence, 56 gradient encoded directions, TR = 9000 ms, TE = 66.2 ms, NEX = 1, FOV = 256 × 256 mm^2^, voxel size = 1 × 1 × 2 mm^3^, 75 axial slices with no gap between slices, flip angle = 90° and b value = 1000 s/mm2.

**Neuroimaging data processing**

*fMRI data processing*

Functional MRI data was processed using AFNI [[2](#_ENREF_12)]. For each subject, the first four volumes of data were discarded to allow for MR signal stabilization. Functional images were motion-corrected and coregistered to the anatomical scan. Coregistration was checked visually. For cases in which coregistration was not acceptable, align_epi_anat.py was used to align the structural T1 images to the functional images. Functional data were smoothed with a 6-mm full-width half maximum (FWHM) Gaussian kernel. Time-series of the functional data from each voxel was scaled to a mean of 100, and AFNI’s 3dDeconvolve was then used to perform a voxel-wise regression analysis with six motion parameters regressed out. This analysis yielded a voxel-wise t-statistic which was then thresholded at t = 4 (p < 0.0001).

*DTI data processing*

DTI data were processed using FSL (v5.0) [[3](#_ENREF_13)]. Data were first corrected for motion (via affine transformation) and eddy current distortion. Next, the gradient direction vectors were corrected for head motion using fdt_rotate_bvecs function provided in FSL. Once all images were registered in a data set, a brain mask was created to exclude extracerebral matter using the BET function. DTI data were then analyzed using the FDTFIT function to calculate the tensor diffusion for each point in the mask and generate results of FA values. The standard white-matter atlas (JHU-ICBM-FA-2mm.nii.gz) was then registered to each patient’s native FA space. Ipsilesional and contralesional PLIC were defined in the standard brain (JHU-ICBM-labels-2mm.nii.gz). Mean FA values within the PLIC from each hemisphere were calculated using 3dROIstats provided in AFNI. In further analysis, mean values of FA and diffusivities were compared in the ipsilesional and contralesional PLIC.

**Passive vs. active motor task**

In our study, all patients underwent two fMRI scans and performed finger tapping with the impaired hand. For patients who were unable to perform the finger tapping task independently, we performed experimenter-assisted passive finger tapping.

Passive motor and sensory tasks have already been successfully applied in small patient groups for therapy monitoring and evaluation during rehabilitation after stroke [4-5]. Blatow et al [6] demonstrated that equal activation levels within identical anatomical locations for passive and active motor tasks.

To examine the effect of passive vs. active motor tasks in our study, we performed sub-group analysis by grouping patients into two groups—passive-task group (n = 5) and active-task group (n = 4). With a longitudinal study design, each patient received multiple neuroimaging and behavioral assessments and this allows for a moderate-size dataset to be utilized for statistical analysis on these two sub-groups. The Wilcoxon rank-sum test was used for group comparison. We did see significant differences in corticomotor activity in terms of voxel counts between passive vs. active finger tapping.

1. Passive tasks showed greater activation in the sensorimotor region than active tasks ipsilesionally (p-value = 0.001) but with no significant differences contralesionally (p-value = 0.44) (Figure e1).
2. Passive tasks also generated significantly greater activation ipsilesionally than contralesionally (Wilcoxon signed-rank test, p-value = 0.018) while active tasks showed no significant differences (p-value = 0.21) in activation between ipsilesional and contralesional areas similar to the overall group (Figure e2).
3. However, there was observed a negative relationship (not significant, GEE p-value = 0.275) between FA and ipsilesional activation in the active-task subgroup (Figure e3) which only became significant (p-value = 0.025) in the overall group (active + passive task) (Figure 7 in main manuscript), suggesting that the overall relationship between DTI and fMRI measures may be irrespective of active or passive task.

Figure e1: Passive vs. Active task: voxel counts compared on ipsilesional (left) and contralesional (right) side for impaired finger tapping.


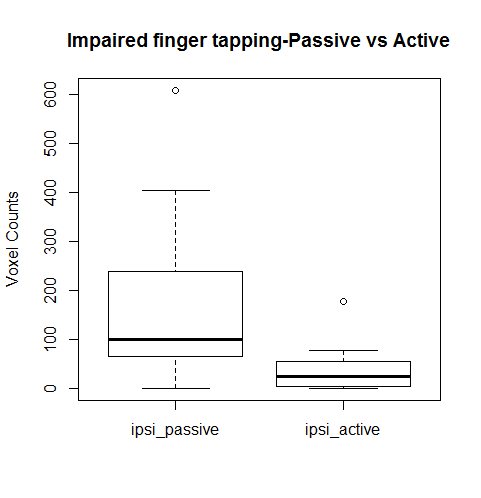

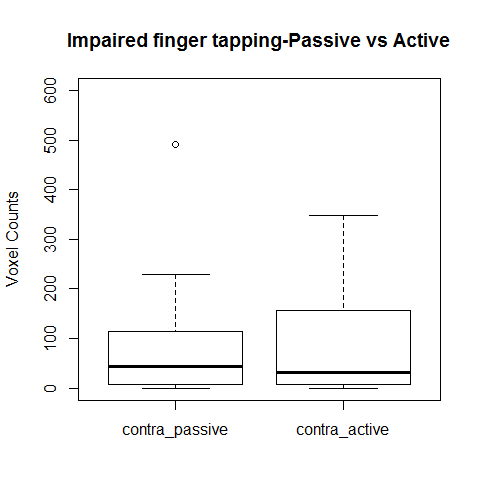


**p = 0.001**

Figure e2: Ipsilesional vs. Contralesional voxel counts compared for passive (left) and active (right) finger tapping tasks.


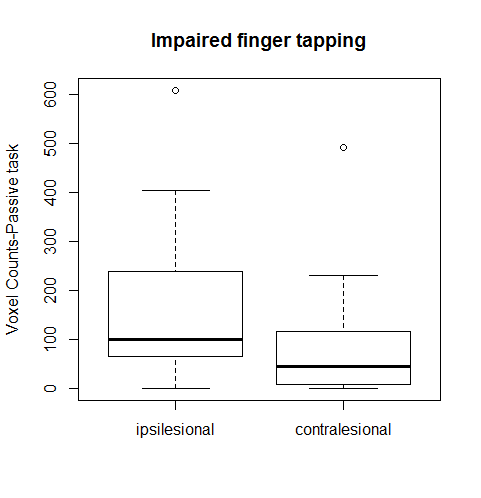

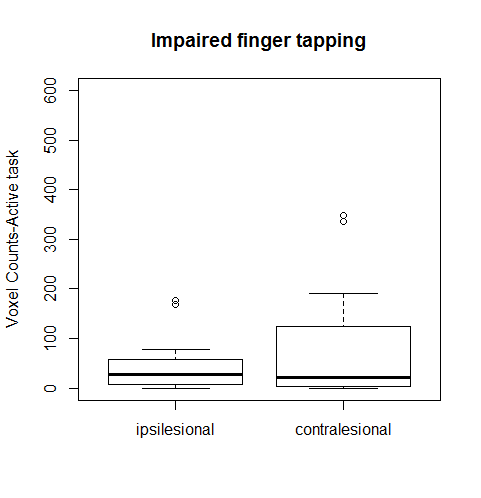


**p = 0.018**

Figure e3: Correlation analysis of fMRI activation (i.e., ipsilesional voxel counts) and DTI measures (i.e., ipsilesional PLIC-FA). Spearman rank correlation coefficient = -0.490, p-value = 0.089; GEE p-value = 0.275


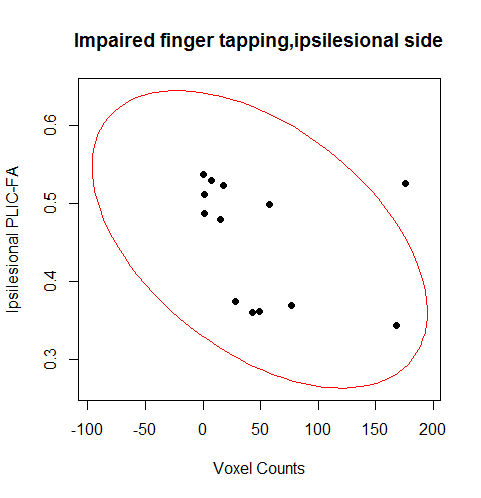


References:

1. Danilov YP, Tyler ME, Skinner KL, Hogle RA, Bach-y-Rita P (2007) Efficacy of electrotactile vestibular substitution in patients with peripheral and central vestibular loss. J Vestibul Res-Equil 17:119-130.

2. Cox RW (1996) AFNI: software for analysis and visualization of functional magnetic resonance neuroimages. Comput Biomed Res 29: 162-173.

3. Smith SM, Jenkinson M, Woolrich MW, et al. (2004) Advances in functional and structural MR image analysis and implementation as FSL. Neuroimage 23:S208-S219.

4. Tombari D, Loubinoux I, Pariente J, et al. (2004) A longitudinal fMRI study: in recovering and then in clinically stable sub-cortical stroke patients. Neuroimage 23:827–839.

5. Ward NS, Brown MM, Thompson AJ, Frackowiak RS. (2006) Longitudinal changes in cerebral response to proprioceptive input in individual patients after stroke: an FMRI study. Neurorehabil Neural Repair 20:398–405.

6. Blatow M1, Reinhardt J, Riffel K, Nennig E, Wengenroth M, Stippich C. (2011) Clinical functional MRI of sensorimotor cortex using passive motor and sensory stimulation at 3 Tesla. J Magn Reson Imaging 34(2):429-37.

**Tables/Figures**

Table S1: Clinical motor outcome assessments of the stroke-affected limb

Figure S1: System design of the rehabilitation device utilizing BCI technology.

Figure S2: Correlation analyses between corticomotor activity and motor outcomes assessed in the impaired hand.

Table S1

| **Groups** | **Subject ID** | **Time points** | **ARAT** | **SIS**  **Hand Function** |
| --- | --- | --- | --- | --- |
| **Experimental Group** | CI001 | Pre-therapy | 0 | 0 |
|  |  | Mid-therapy | 3 | 0 |
|  |  | Post-therapy | 0 | 0 |
|  |  | 1-month-post-therapy | 0 | 0 |
|  | CI002 | Pre-therapy | 0 | 0 |
|  |  | Mid-therapy | 0 | 0 |
|  |  | Post-therapy | 0 | 0 |
|  |  | 1-month-post-therapy | 0 | 0 |
|  | CI003 | Pre-therapy | 57 | 40 |
|  |  | Mid-therapy | 57 | 55 |
|  |  | Post-therapy | 57 | 70 |
|  |  | 1-month-post-therapy | 57 | 75 |
|  | CI004 | Pre-therapy | 3 | 0 |
|  |  | Mid-therapy | 0 | 0 |
|  |  | Post-therapy | 0 | 0 |
|  |  | 1-month-post-therapy | 0 | 0 |
|  | CI005 | Pre-therapy | 56 | 50 |
|  |  | Mid-therapy | 46 | 70 |
|  |  | Post-therapy | 54 | 50 |
|  |  | 1-month-post-therapy | 57 | 57.5 |
| **Control**  **Group** | CT001 | Control-time-point-1 | 0 | 0 |
|  |  | Control-time-point-2 | 0 | 0 |
|  |  | Control-time-point-3 | 0 | 0 |
|  |  | Pre-therapy | 0 | 5 |
|  |  | Mid-therapy | 0 | 0 |
|  |  | Post-therapy | 0 | 0 |
|  |  | 1-month-post-therapy | 0 | 0 |
|  |  | Control-time-point-1 | 54 | 55 |
|  | CT002 | Control-time-point-2 | 57 | 35 |
|  |  | Control-time-point-3 | 51 | 50 |
|  |  | Pre-therapy | 53 | 75 |
|  |  | Mid-therapy | 57 | 75 |
|  |  | Post-therapy | 54 | 75 |
|  | CT003 | Control-time-point-1 | 26 | 10 |
|  |  | Control-time-point-2 | 27 | 0 |
|  |  | Control-time-point-3 | 32 | 20 |
|  |  | Pre-therapy | 27 | 10 |
|  |  | Mid-therapy | 28 | 30 |
|  |  | Post-therapy | 40 | 35 |
|  |  | 1-month-post-therapy | 43 | 45 |
|  |  | Control-time-point-1 | 54 | 30 |
|  | CT004 | Control-time-point-2 | 57 | 45 |
|  |  | Control-time-point-3 | 57 | 45 |
|  |  | Mid-therapy | 47 | 45 |

Figure S1

Figure S2: Correlation analyses between corticomotor activity (evaluated as active voxel counts) and motor outcomes (ARAT and SIS-Hand function) assessed in the impaired hand. Spearman rank correlation tests showed significant relationships between task fMRI and motor outcome measurements.


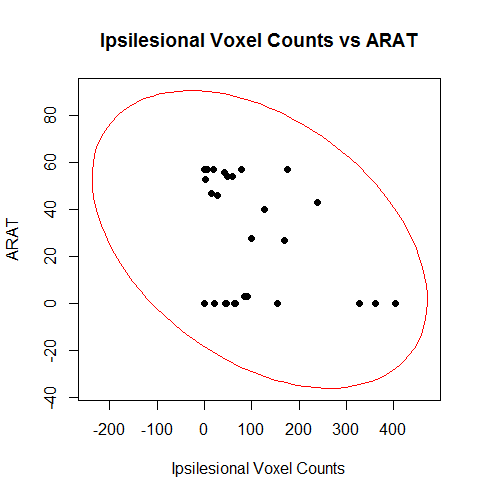


ARAT

Voxel Counts vs. ARAT (ρ = -0.414; p-value = 0.026)


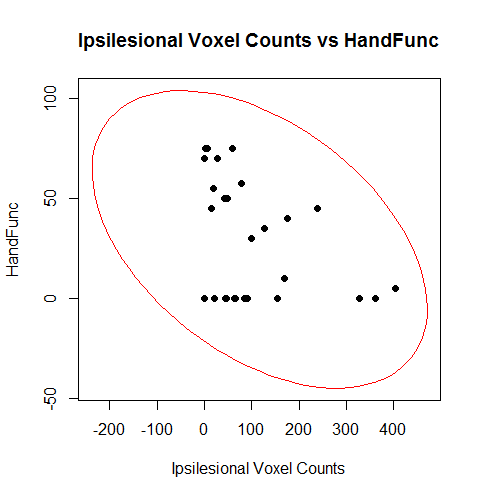


SIS-Hand

Voxel Counts vs. SIS-Hand (ρ = -0.463; p-value = 0.012)
